# Quorum-sensing-regulated virulence factors in *Pseudomonas aeruginosa* are toxic to *Lucilia sericata* maggots

**DOI:** 10.1099/mic.0.032730-0

**Published:** 2010-02

**Authors:** A. S. Andersen, B. Joergensen, T. Bjarnsholt, H. Johansen, T. Karlsmark, M. Givskov, K. A. Krogfelt

**Affiliations:** 1 Statens Serum Institut ABMP, Artillerivej 5, Copenhagen S, Denmark; 2 Copenhagen Wound Healing Center, Bispebjerg Hospital, Bispebjerg bakke 23, Copenhagen NV, Denmark; 3 University of Copenhagen Faculty of Health Sciences, Blegdamsvej 3B, 2200 Copenhagen N, Denmark

## Abstract

Maggot debridement therapy (MDT) is widely used for debridement of chronic infected wounds; however, for wounds harbouring specific bacteria limited effect or failure of the treatment has been described. Here we studied the survival of *Lucilia sericata* maggots encountering *Pseudomonas aeruginosa* PAO1 in a simple assay with emphasis on the quorum-sensing (QS)-regulated virulence. The maggots were challenged with GFP-tagged *P. aeruginosa* wild-type (WT) PAO1 and a GFP-tagged *P. aeruginosa* Δ*lasR* *rhlR* (ΔRR) QS-deficient mutant in different concentrations. Maggots were killed in the presence of WT PAO1 whereas the challenge with the QS mutant showed a survival reduction of ∼25 % compared to negative controls. Furthermore, bacterial intake by the maggots was lower in the presence of WT PAO1 compared to the PAO1 ΔRR mutant. Maggot excretions/secretions (ES) were assayed for the presence of QS inhibitors; only high doses of ES showed inhibition of QS in *P. aeruginosa*. Thus *P. aeruginosa* was shown to be toxic to *L. sericata* maggots. This, coupled to the preferential feeding by the maggots and reduced ingestion of *P. aeruginosa*, could explain MDT failure in wounds colonized by *P. aeruginosa.* Wounds heavily colonized with *P. aeruginosa* should be a counterindication for MDT unless used in combination with a pre-treatment with other topical therapeutics targeting *P. aeruginosa*.

## INTRODUCTION

Maggot debridement therapy (MDT) of chronic wounds is a controlled application of commercially produced sterile larvae from the greenbottle fly *Lucilia sericata*. The application of maggots in a clinical setting is performed either in sealed nylon pouches/bags that allow fluid and gas exchange with the wound environment (Biobags), or as a free-range version of the therapy, where the maggots are applied directly to the wound bed, sealed in with a covering net and dressing (Sherman *et al.*, 2000). The treatment modality is a standard procedure at wound care centres all over the world and is a safe therapeutic option. Many patients with devitalized and necrotic chronic wounds are ideal candidates for MDT. The maggots gently and thoroughly remove necrotic tissue by mechanical action and by extracorporeal proteolytic digestion during a 3–5 day application, after which they are rinsed from the wound or the bag is simply removed, leaving the healthy granulation tissue unharmed. The maggots are additionally believed to secrete antimicrobial compounds into the wound, kill ingested bacteria in their gut and alkalinize the wound (Mumcuoglu *et al.*, 2001; Sherman *et al.*, 2000; Thomas *et al.*, 1999), and their secretions inhibit multiple neutrophil and monocyte pro-inflammatory responses (van der Plas *et al.*, 2007, 2009) – all effects beneficial in order to heal a chronic wound. However, a few reports have stated that MDT in some cases facilitates an infection, especially when specific bacterial species are present in the wound prior to treatment (Fleischmann *et al.*, 2004; Fleischmann, 2007). MDT has also been observed to fail in some cases due to the death of the maggots in the wound environment or to low feeding activities of the maggots (Fleischmann, 2007; Renner *et al.*, 2008).

A major beneficial effect of MDT is the reduction of the wound bio-burden. The bactericidal effect of maggot excretions/secretions has been described against a broad range of Gram-positive micro-organisms, including meticillin-resistant *Staphylococcus aureus* (Bexfield *et al.*, 2008; Bowling *et al.*, 2007; Cazander *et al.*, 2009a), but they are less effective against Gram-negative bacteria, in particular *Pseudomonas aeruginosa* (Cazander *et al.*, 2009a; Jaklic *et al.*, 2008; Kerridge *et al.*, 2005; Steenvoorde & Jukema, 2004). Chronic wounds harbour mixed microbial communities (Howell-Jones *et al.*, 2005) and in particular it has been shown that *P. aeruginosa* increases the wound size when colonizing chronic venous leg ulcers (Gjõdsbõl *et al.*, 2006). Wounds are in general susceptible to infection due to the development of microbial communities within and around the wound environment (Bowler, 2003), and can act as reservoirs of antibiotic resistant micro-organisms in hospitals and nursing homes (Day & Armstrong, 1997). It is suggested that bacteria during colonization of chronic wounds are growing in microcolonies also known as biofilms (Bjarnsholt *et al.*, 2008; Davis *et al.*, 2008; James *et al.*, 2008; Kirketerp-Moller *et al.*, 2008; Serralta *et al.*, 2001).

Biofilms and the chemically based bacterial cell communication denoted quorum sensing (QS) play important roles in a multitude of human infections (Costerton *et al.*, 1999). In *P. aeruginosa,* the QS system is divided into two hierarchically ordered systems termed *las* and *rhl*, with the *las* system positioned higher in the signal cascade. Both systems consist of a specific pair of genes, *lasI/lasR* and *rhlI/rhlR* respectively (Pesci & Iglewski, 1997). The *I*-genes encode synthases that synthesize either 3-oxo-dodecanoylhomoserine lactone (3-oxo-C12-HSL) in the *las* system or butyrylhomoserine lactone (C4-HSL) in the *rhl* system. The *R* genes encode regulatory proteins which, guided by the abundance and density of C4-HSL and 3-oxo-C12-HSL, activate gene expression of numerous target genes including expression of virulence factors, such as elastase, (alkaline) proteases, rhamnolipids, pyocyanin and cyanide (Pearson *et al.*, 1997). Interconnecting the systems in the QS hierarchy is the quinolone signal (PQS) system that cooperatively regulates expression of *rhlI* and *lasB* (Diggle *et al.*, 2003). Several opportunistic pathogenic bacteria such as *P. aeruginosa* and *S. aureus* show QS-mediated organization (Costerton *et al.*, 1999; Qazi *et al.*, 2006; Rasmussen & Givskov, 2006), thus increasing speculation regarding the involvement of QS and biofilm formation in the chronicity of wounds.

An exotoxin of *Pseudomonas fluorescens* has been shown to be toxic to larvae and pupae of the domestic house fly *Musca domestica* (Padmanabhan *et al.*, 2005), although the metabolite and mode of action are not known. Furthermore, maggot excretions/secretions are highly effective against *S. aureus* biofilms *in vitro* but less effective against *P. aeruginosa* biofilms (van der Plas *et al.*, 2008).

All the above factors inspired us to design a solid-medium assay for assessing the effect of bacterial challenges on maggot survival *in vitro* in order to investigate the possible role of QS-controlled virulence factors in *P. aeruginosa* as a cause of MDT failure.

## METHODS

### Maggots and maggot secretions.

In order to mimic the patient situation with respect to logistics and larval life stages at application in the wound, larvae were purchased through and delivered to the Copenhagen Wound Healing Centre (CWHC) from a commercial supplier (BioMonde or Zoobiotic) via the usual procedures at the CWHC. Upon delivery, vials of sterile first-instar larvae of *L. sericata* were used for initiation of the experiments within a timeframe of 2 h. Additionally, maggot excretions/secretions (ES) were collected from 300 aseptically reared first-instar maggots (ES1) and from 400 aseptically reared and actively feeding second–third-instar maggots (ES2), approximately 5 g wet weight. The maggots used for ES collection were a kind gift from Dr Alun Morgan of Zoobiotic Ltd, Bridgend, UK. Maggots were added to a sterile container, supplemented with 200 μl sterile MilliQ water per g of maggots and incubated at 30 °C for 60 min in the dark; the surplus fluid was then siphoned off and centrifuged at 1300 ***g*** for 5 min to remove particulate matter. The supernatant was checked for sterility and stored at −20 °C (van der Plas *et al.*, 2008). Total protein concentration of ES was determined with the Pierce Micro BCA protein assay kit (Fisher Thermo Scientific) according to the manufacturer's instructions.

### Micro-organisms.

The wild-type (WT) *P. aeruginosa* was obtained from the Pseudomonas Genetic Stock Center (http://www.pseudomonas.med.ecu.edu; strain PAO0001, hereafter PAO1). The *P. aeruginosa* Δ*lasR rhlR* mutant (ΔRR) (Bjarnsholt *et al.*, 2005) was constructed using previously described knockout systems (Beatson *et al.*, 2002). This isolate has served as DNA source for the Pseudomonas Genome Project (http://www.pseudomonas.com) and subsequently as template for the design of the *P. aeruginosa* GeneChip (Affymetrix). The knockout mutants were verified by Southern blot analysis and by screening for acylhomoserine lactone production (QS signals). A stable green fluorescent protein (GFP) constitutively expressed on plasmid pMRP9 (Davies *et al.*, 1998) was used to tag the bacteria. PAO1 carrying the plasmid pMHLB, containing a transcriptional fusion of the LasR-regulated *lasB* promoter and the gene for an unstable version of GFP, was used for the LasR inhibition assay (Hentzer *et al.*, 2002). A similar strain for the RhlR inhibition assay was used, the only difference being a replacement of the *lasB* : : *gfp* construct on the pMHLB plasmid with a *rhlA* : : *gfp* transcriptional fusion (Yang *et al.*, 2009).

### Survival assay of larvae on plates.

The larvae were aseptically transferred onto 9 cm 5 % blood agar Petri dishes (SSI art. nr 677) (hereafter referred to as plates), treated with five different preconditions and each covered with a gas-permeable sterile pre-cut piece of Tegapore wound contact material (3M) tightly taped to the top of the plates. This seal allowed ventilation and prevented escape of the larvae. Precondition 1 (control plates): plates were incubated 17 h overnight (O/N) at 37 °C prior to the assay. Precondition 2 (WT low): 6×10^2^ c.f.u. WT PAO1 bacteria were spread on the plates and incubated O/N at 37 °C prior to the assay, resulting in dispersed growth of single colonies of bacteria. Precondition 3 (WT high): 6×10^5^ c.f.u. WT PAO1 bacteria were spread on the plates and incubated O/N at 37 °C prior to the assay, resulting in a dense lawn of bacteria. Precondition 4 (ΔRR low): 6×10^2^ c.f.u. ΔRR PAO1 bacteria were spread on the plates and incubated O/N at 37 °C prior to the assay, resulting in dispersed growth of single colonies of bacteria. Precondition 5 (ΔRR high): 6×10^5^ c.f.u. ΔRR PAO1 bacteria were spread on the plates and incubated O/N at 37 °C prior to the assay, resulting in a dense lawn of bacteria. Plates similar to preconditions 2 and 3 were additionally supplemented to total concentrations of 10 μM (precondition 6) and 75 μM (precondition 7) with the known quorum-sensing-inhbiting (QSI) compound furanone C30 (Hentzer *et al.*, 2003a) prior to inoculation in order to synthetically induce a phenotype similar to that of ΔRR PAO1. Ten sterile first-instar maggots were added per plate for the different preconditions (*n*=100 for preconditions 1–5 and *n*=30 for preconditions 6 and 7) and incubated at 37 °C in the dark.

Maggot survival was monitored at 2 h intervals for the first 70 h and subsequently checked after 90 h (at assay termination), mimicking the 3–4 days duration of MDT in the clinic. Maggot survival was monitored by removal of immobile/inactive maggots into sterile Petri dishes dedicated to each assay plate and recorded. Maggot death was verified by circling the immobile maggots with a permanent marker and the observation of no movement for 4 h. In the case of movement maggots were reintroduced into the assay plate from which they had been removed. Maggots contained in 2×2 cm biobags (BioMonde) (50 maggots per bag) were also challenged on the WT PAO1 high and ΔRR PAO1 high assay plates independently of the other experiments. The bags were allowed to lie on top of the inoculated plates for 90 h and the clearing zone beneath the bags was monitored. Maggot viability was observed after 90 h, but it was not monitored during the assay since this estimate was impossible to perform through the bags.

Maggot survival was analysed by Kaplan–Meier survival plots using GraphPad Prism Version 4.0 (GraphPad Software Inc.). Comparison of survival plots was performed using a logrank test.

### Larvae bacterial feeding assay.

Five first-instar maggots per plate were added to three plates with either the WT high precondition or the ΔRR high precondition and allowed to forage for 20 min. The maggots were then transferred to sterile Petri dishes and briefly kept at −20 °C for 3 min in order to slow down their activity level, after which they were photographed in snapshot series of 20 pictures per maggot using a fluorescence microscope. A representative image from the anterior part of each maggot containing the larval crop from each setup was selected and evaluated visually. Crop fluorescence was used as an indication of the amount of food uptake by the maggots when compared to the minimal auto-fluorescence from maggots taken from plates without bacteria. All microscope observations and image acquisitions were performed with a Zeiss LSM 510 CLSM equipped with lasers, detectors and filter sets for monitoring of green fluorescence. Images were obtained using a 10×/1.3 objective.

### Larvae bacterial chemotaxis assay.

Four sterile first-instar maggots were added to 1-day-old c.f.u. spot-plates containing either ΔRR PAO1 or WT PAO1 in spots of increasing bacterial density and areas of plate not containing bacteria, thereby presenting the maggots with movement preference options. Plates were incubated for 2 h at 37 °C and inspected with respect to the maggot's movement as revealed by tracks in the bacterial spots. Experiments were performed in triplicate.

### QS inhibition.

Fifteen third-instar maggots taken from the precondition 1 plates after 90 h were added to duplicate sets of three 1.5 ml tubes containing 1 ml ethyl acetate (Full 1), 1 ml methanol (Full 2), or 1 ml sterile MilliQ water (Full 3), respectively, and homogenized with a sterile plastic pestle. Full maggot samples were cleared of particulate matter by centrifugation and 25 μl of the resulting extract was tested for the presence of general QSI compounds along with 25 μl of the collected ES using the QSIS1 selector assay as described by Rasmussen *et al.* (2005). ES1 and ES2 were also tested, in concentrations ranging from 0.5 to 400 μg total protein, for the ability to inhibit *lasB* and *rhlA* in *P. aeruginosa in vitro,* as described by Yang *et al.* (2009). To establish the presence of a dose–response relationship to ES1 and ES2, twofold serial dilutions were made with growth medium (ABT with 0.5 %, w/v, Casamino acids and 0.5 % glucose) in a microtitre dish. Each well contained 150 μl ES solution (diluted). Subsequently, 150 μl of an overnight culture (diluted 1 : 100) of either *P. aeruginosa* PAO1 *lasB-gfp*(ASV) or *P. aeruginosa* PAO1 *rhlA-gfp*(ASV) was added. Growth was monitored as OD_450_ over a time-course of 16 h, and GFP expression was measured (excitation/emission wavelength 485/535 nm respectively).

## RESULTS

### Maggot survival

**Fig. 1. f1:**
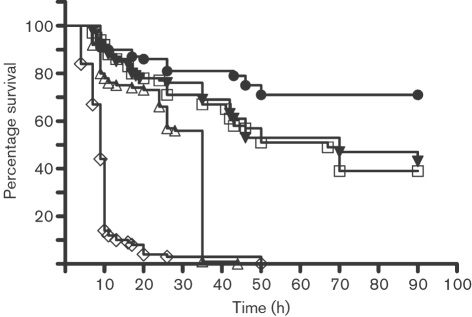
Kaplan–Meier survival plots for the five precondition treatments (*n*=100 for each precondition). Curves were tested with a logrank test as to whether they were significantly different. All survival plots were significantly different from the control (•) (*P*<0.0001) and all plots were individually significantly different from each other (*P*<0.0001) except for the comparison between the ΔRR high precondition (▾) and the ΔRR low precondition (□), which were not significantly different. ◊, WT high precondition; ▵, WT low precondition.

A novel plate assay was developed in order to establish whether QS-dependent virulence factors are detrimental to the maggots. To monitor maggot survival *in vitro* WT PAO1 and ΔRR PAO1 strains were used. In Fig. 1, the Kaplan–Meier survival analysis of the maggots shows that the presence of WT PAO1 in both the high and low dose severely impaired maggot survival compared to the control plates, with almost 0 % survival for the maggots subjected to the WT high precondition after 20 h.

A similar final mortality was seen for the WT low precondition, but with a 15 h lag time compared to the WT high precondition. The logrank test showed that all survival curves were significantly different from the control curve (*P*<0.0001) and all curves were individually significantly different from each other (*P*<0.0001), except for the comparison between the ΔRR high precondition and the ΔRR low precondition. This confirmed our suspicion that QS-controlled virulence factors in part were causing impaired survival of the maggots. Comparable results were seen for the maggots confined in Biobags (results not shown).

### Maggot feeding and bacterial ingestion

**Fig. 2. f2:**
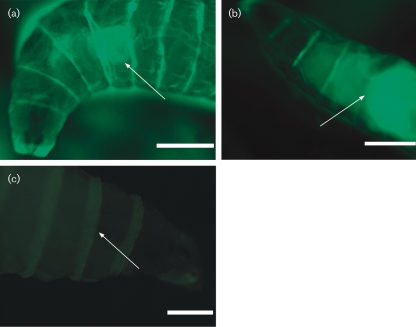
(a, b) Representative pictures of the anterior part of maggots having foraged on a plate with the WT high precondition (a) or the ΔRR high precondition (b). (c) Representative overexposed picture (5 s) of the anterior part of a maggot having foraged on a negative control plate without bacteria, indicating minimal background fluorescence. Arrows indicate crop position. Scale bars, 0.5 mm.

In order to explore other possibilities besides the QS-controlled toxicity, we aimed to use scanning confocal laser microscopy to visualize the ingested bacteria within the maggots via the GFP tag. As shown in Fig. 2, the maggots that foraged on the plates with high numbers of WT PAO1 showed little fluorescence in the anterior part of the body, corresponding to the crop where the maggots initially process ingested food. This was in marked contrast to maggots from plates with ΔRR PAO1 in high numbers, where massive fluorescence was observed in the anterior part of the maggots corresponding to the location of the crop. Technical problems during fixation of maggots prevented us from getting quantitative measurements of the actual fluorescence intensity, so the data are representative images from the WT high, ΔRR PAO1 high and control plates. The data strongly indicate a reduced bacterial uptake by the maggots on the WT high plates. The high intake of ΔRR PAO1 compared to the WT PAO1 could suggest that the presence of *P. aeruginosa* actively expressing QS-controlled virulence factors leads to reduced food intake by *L. sericata* maggots.

### QS-controlled factors influence maggot chemotaxis

**Fig. 3. f3:**
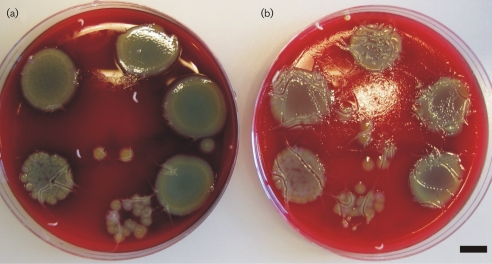
(a) WT PAO1 c.f.u. spot-plate with four maggots. (b) ΔRR PAO1 c.f.u. spot-plate with four maggots. Both plates were incubated in the dark for 120 min at 37 °C. Maggot movement is indicated by tracks on the plates and is reduced on (a) as compared to (b). Scale bar, 1 cm.

As a preliminary indicative experiment to study whether maggots will actively move away from high concentrations of QS-producing *P. aeruginosa*, a simple experiment was carried out on spot-plates used for the c.f.u. determinations for the precondition plates. As shown in Fig. 3, the maggots seemed to actively avoid the areas with the high concentrations of WT PAO1 as compared to the same concentrations of the ΔRR PAO1. This is comparable to observations from the survival assays, where the maggots from the WT PAO1 plates were recovered dead positioned on the Tegapore seal, indicating that the maggots will avoid high concentrations of WT PAO1.

### QS inhibitors in maggot extracts and ES

**Fig. 4. f4:**
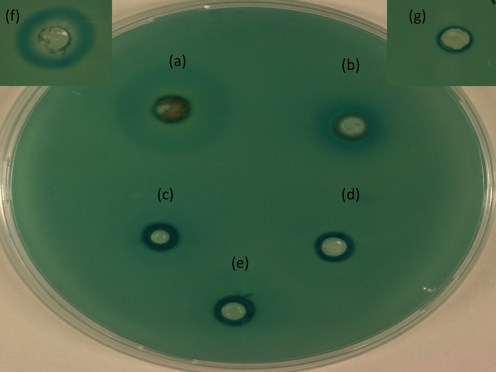
(a–e) QSIS1 assay: (a) 25 μl ethyl acetate full maggot extract, (b) 25 μl methanol full maggot extract, (c) 25 μl MilliQ water full maggot extract, (d) 150 μg ES1, (e) 150 μg ES2. (f) Positive control: 1 mM patulin. (g) Negative control: 25 μl 10 % methanol.

Maggot ES and full maggot extracts were tested using the QSIS1 system and specifically for *P. aeruginosa* QSI activity. As seen in Fig. 4(a, b), the addition of the ethyl acetate and methanol extracts from the full maggots showed a general QSI activity, but this was slight as compared to the positive control patulin shown in Fig. 4(f). ES1 and ES2 did not appear to convey any QSI effect in the QSIS1 assay Fig. 4(d, e).

**Fig. 5. f5:**
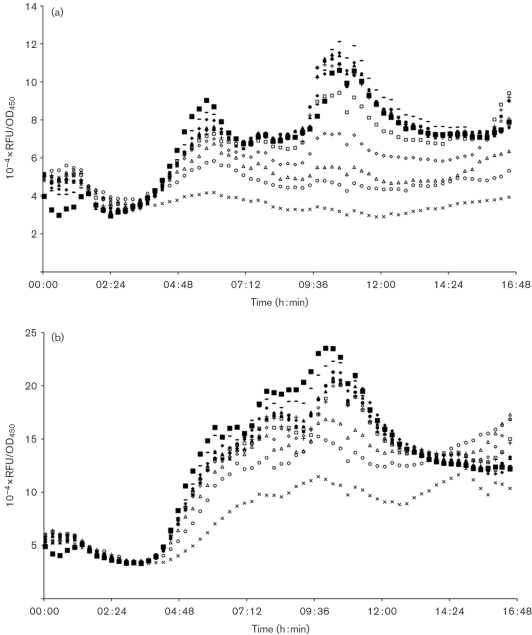
Dose–response curves of the effect of ES2 on *P. aeruginosa* QS using QS monitors. (a) PAO1 (*rhlA-gfp*); (b) PAO1 (*lasB-gfp*). ES2 added: ×, 400 μg; ○, 200 μg; ▵, 100 μg; ◊, 50 μg; □ 25 μg; +, 12.5 μg; •, 8 μg; ▴, 4 μg; ⧫, 2 μg; -,1 μg; ▪, blank. Growth and fluorescence were followed over time and are depicted as Gfp/OD_450_ curves. RFU, relative fluorescence units.

As seen in Fig. 5(a) maggot ES seemed to dose-dependently inhibit PAO1 *rhlA* expression, but they inhibited PAO1 *lasB* expression to a lesser extent (Fig. 5b[Fig f5]). Addition of ES2, even in the highest concentration of 400 μg, to the PAO1 (*rhlA-gfp*) and PAO1 (*lasB-gfp*) QS monitoring assays did not show a bactericidal effect against *P. aeruginosa* (results not shown), which is in line with previous reports on the antimicrobial activity of ES (Cazander *et al.*, 2009a; Jaklic *et al.*, 2008; Kerridge *et al.*, 2005; Steenvoorde & Jukema, 2004).

## DISCUSSION

In order to test the *in vitro* effect of bacterial wound pathogens upon survival of maggots of the blow fly *L. sericata*, a simple assay was developed. The assay was used to elicit an explanation for the treatment failure seen during MDT for the debridement of chronic wounds colonized/infected with *P. aeruginosa*. In the majority of cases MDT is a safe therapeutic option for debridement of otherwise very difficult to treat cases of chronic wounds.

Maggot survival is affected by transport and storage conditions (Zhang *et al.*, 2008), here reflected in our assay, which did not confer 100 % survival of the maggots on the control plates (Fig. 1).

The slower onset of maggot mortality resulting from the WT PAO1 high compared to the WT PAO1 low condition may in part be explained by the observed reduced food uptake (Fig. 2) coupled to the preferential movement of the maggots away from areas of the assay plates harbouring WT PAO1 (Fig. 3). Thus WT PAO1 in the low precondition plates accumulates to critical mass/toxicity when unchallenged by the maggots, eventually resulting in 100 % mortality. The reduced survival of the maggots seemed to be associated with the expression of QS-controlled virulence factors by the bacteria, as an increase in survival was seen in both ΔRR PAO1 high and low preconditions, in which the bacteria do not express QS-dependent virulence factors. The observed impairment of maggot survival in the ΔRR PAO1 high and low conditions as compared to the negative controls may be attributed to non-QS-controlled bacterial metabolites. However, the survival and nutritional status of the maggots on the ΔRR precondition plates was, as shown in Figs 1 and 2, significantly improved compared to that on the WT precondition plates. In order to rescue the maggots a QSI compound, furanone C30, was supplemented to plates, but it did not sufficiently inhibit the WT PAO1 effect. The solid-media assay developed here does not allow the continuous supplementation that is required for a sufficient QSI effect (Hentzer *et al.*, 2003b). Since activities of the maggots in MDT take place on wound surfaces, performing the experiments in liquid media was not considered an alternative.

Our results indicate that the problems that clinicians experience with *P. aeruginosa* and MDT are caused by *P. aeruginosa* QS-controlled toxicity to the maggots. In addition, the failure of maggots to eliminate *P. aeruginosa* due to preferential feeding may thereby create or expand the wound micro-niches that exist within chronic wounds (Andersen *et al.*, 2007; Fazli *et al.*, 2009; Kirketerp-Moller *et al.*, 2008). Therefore, wounds heavily colonized with *P. aeruginosa* should be a counterindication for MDT or should be treated with a substantially higher number of maggots or more frequent dressing/maggot changes (Steenvoorde & Jukema, 2004; van der Plas *et al.*, 2008). However, adding more maggots should be weighed against the added costs of this treatment regime as opposed to supplementing or pre-treating the wound with other topical therapeutics.

The QSIS1 assay of full maggot extracts in different solvents showed a limited QSI activity. ES did, at high concentrations, inhibit PAO1 *rhlA* expression and to a lesser extent PAO1 *lasB*, which indicated that components within ES at these concentrations interfere with the QS-controlled induction of *lasB*. However, since 400 μg in this limited volume is in the upper part of the therapeutic range of ES that can be achieved locally in wounds during MDT (van der Plas *et al.*, 2008), this inhibition is not clinically relevant, especially in heavily colonized wounds. Comparable results were seen for both ES1 and ES2. ES in concentrations ranging from 0.2 to 200 μg did not mimic or antagonize the actions of various short- and long-chained *N*-acylhomoserine lactones, even though addition of ES led to *S. aureus* and *P. aeruginosa* biofilm breakdown *in vitro* (Cazander *et al.*, 2009b; van der Plas *et al.*, 2008). However, ES in concentrations ranging from 100 to 400 μg in our study seemed to inhibit expression of *lasB*, even though it did not abrogate it. The mechanisms and compounds within ES leading to the reduced expression of *lasB* in this study will be the subject of further investigations.

A study by Stoltz *et al.* (2008) elegantly showed that using transgenic paraoxonase-1 (PON1)-expressing *Drosophila melanogaster* flies rescued the flies in an abdominal infection model from *P. aeruginosa*-induced lethality. The reduced virulence of *P. aeruginosa* was dependent upon enzymic lactonase activity of PON1 not normally expressed in *D. melanogaster.* PON1 degrades and disrupts the small autoinducer acylhomoserine lactone QS molecules (Ozer *et al.*, 2005). These findings support the lethality observed in our study due to *P. aeruginosa*. Although the study by Stoltz *et al.* (2008) was performed in a different insect, *D. melanogaster*, and with direct infection of adult flies, toxicity of *P. aeruginosa* showed QS dependence. In our study the mere presence of large quantities of WT *P. aeruginosa* without any predisposition of the maggots via chemical or physical challenge was toxic to the maggots. A clinical implication of these results is that wounds heavily colonized with *P. aeruginosa* should be a counterindication for MDT unless used in combination with a pre-treatment with other topical therapeutics targeting *P. aeruginosa*. The specific QS-controlled *P. aeruginosa* virulence factors involved in the reduced maggot survival will be the subject of further research.

## Abbreviations

- ES, excretions/secretions
- MDT, maggot debridement therapy
- QS, quorum sensing
- QSI, quorum sensing inhibiting
- SSI, Statens Serum Institut
- PON1, paraoxonase-1
